# Reverse genetics approach for arteriviruses using circular polymerase extension reaction

**DOI:** 10.1099/acmi.0.001181.v3

**Published:** 2026-05-21

**Authors:** Rabab T. Hassanien, Wellesley Dittmar, Udeni B. R. Balasuriya, GuanQun Liu, Côme J. Thieulent, Samah Eid, Mariano Carossino

**Affiliations:** 1 Department of Pathobiological Sciences, School of Veterinary Medicine, Louisiana State University, Baton Rouge, LA, USA; 2 Virology Department, Animal Health Research Institute, Agriculture Research Center (ARC), Dokki, Giza 12618, Egypt; 3 Department of Microbiology & Immunology, McGill University, Montreal, Canada; 4 Reference Laboratory for Veterinary Quality Control on Poultry Production, Animal Health Research Institute, Agriculture Research Centre (ARC), Giza 12618, Egypt; 5 Louisiana Animal Disease Diagnostic Laboratory, School of Veterinary Medicine, Louisiana State University, Baton Rouge, LA, USA

**Keywords:** cDNA clones, circular polymerase extension reaction (CPER), equine arteritis virus (EAV), porcine reproductive and respiratory syndrome virus (PRRSV), reporter viruses, reverse genetics

## Abstract

The circular polymerase extension reaction (CPER) has emerged as a high-fidelity, versatile and rapid tool for generating infectious cDNA clones of positive-sense, single-stranded RNA viruses. Here, we implemented this strategy to generate infectious cDNA clones of two closely related members of the family *Arteriviridae*: equine arteritis virus KY84 strain (EAV KY84) and porcine reproductive and respiratory syndrome virus VR2332 strain (PRRSV VR2332). Overlapping cDNA fragments spanning the entire viral genomes were generated and assembled with a linker sequence incorporating critical expression elements, yielding a circularized, full-length viral cDNA genome ready for direct transfection into permissive cells. Following transfection, infectious rEAV KY84 and rPRRSV VR2332 strains were recovered and characterized. Similarly, we generated reporter viruses by inserting the mCherry gene downstream of the nsp1/nsp2 cleavage junction in EAV KY84 ORF1a and the GFP gene downstream of ORF1b of PRRSV VR2332. All CPER-generated viruses showed high identity to their parental strains by next-generation sequencing. Growth kinetics of CPER-generated non-reporter and rEAV KY84-RFP mCherry viruses were comparable to their parental strains; however, the reporter rPRRSV VR2332-GFP showed a significant reduction in viral titres despite its stability for at least 15 passages. In contrast, mCherry expression from rEAV KY84-mCherry was negligible at passage 15 and associated with the deletion of the reporter gene. Taken together, the CPER-based method can be used as a rapid tool to generate infectious cDNA clones of arteriviruses, allowing the easy generation of mutants or reporter viruses that can facilitate the investigation of viral gene functions and their interactions with the host.

## Data Summary

GenBank accession nos.: MG137429, PX841640, PX849429, PX849430, PX849431 and PX849432.

## Introduction

Equine arteritis virus (EAV) (*Alphaarterivirus equid*) and porcine reproductive and respiratory syndrome virus (PRRSV) (*Betaarterivirus americense* and *Betaarterivirus europensis*) are members of the genera *Alphaarterivirus* and *Betaarterivirus*, family *Arteriviridae*, order *Nidovirales* [1,2]. EAV and PRRSV are enveloped viruses with a positive-sense, single-stranded RNA (+ssRNA) genome [3,4]. Both viruses are globally distributed and are among the most economically important viruses in this family. EAV and PRRSV are host-restricted to members of the *Equidae* and *Suidae* families, respectively, and share many biological and molecular properties, including virion morphology, genome organization and replication strategies, as well as a unique set of structural proteins. Both cause reproductive and respiratory diseases and can establish persistent infections in their hosts [5].

EAV is the causative agent of equine viral arteritis, a respiratory, reproductive and systemic disease of horses responsible for outbreaks that cause significant economic losses to the equine industry, primarily associated with the occurrence of widespread abortions in pregnant mares, neonatal mortality and establishment of persistent infection in stallions, leading to interruption of breeding programmes and cancellation of equestrian events [5,8]. The EAV genome is ~12.7 kb in length and contains at least 10 known open reading frames (ORFs) [9]. Approximately two-thirds of the viral genome comprises ORF1a and ORF1b, located at the 5′-end of the genome, which encode two replicase polyproteins (pp1a and pp1ab). These two replicase polyproteins are responsible for encoding at least 13 nonstructural proteins (nsp1–12, including nsp7 *α*/*β*) [7]. The remaining eight ORFs (2a, 2b, 3, 4, 5a, 5b, 6 and 7) are located at the 3′-end of the genome and encode for the structural proteins of the virus (E, GP2, GP3, GP4, ORF5a protein, GP5, M and N, respectively) [9]. The major envelope proteins (M and GP5) form a disulphide-linked heterodimer, while the minor envelope glycoproteins (GP2, GP3 and GP4) form a heterotrimer in the viral envelope [10,15].

PRRSV is the causative agent of PRRS, which presents a complex clinical spectrum. The disease is predominantly characterized by reproductive disorders including abortions, stillbirths, premature births and infertility, along with systemic signs such as anorexia, coughing, pyrexia and respiratory distress. In severe instances, PRRSV infection can lead to mortality. Historically, PRRSV infections have been associated with high mortality rates in young pigs. However, the emergence of highly pathogenic PRRSV strains has shifted the epidemiological landscape, resulting in outbreaks with significant mortality in adult swine populations [16,18]. PRRSV also establishes persistent infections in lymphoid tissues (e.g. tonsils and lymph nodes) as well as in the reproductive tract of the boar [5,19,21]. Despite major efforts for prevention, control and elimination of PRRSV, the virus remains one of the most costly infections affecting the swine industry in the USA and other countries worldwide [22], with estimated annual losses of $1.2 billion USD [23].

The PRRSV genome has a similar organization to that of EAV [5]. It is ~15 kb and contains at least 10 ORFs, including ORF1a, ORF1b, ORF2a, ORF2b, ORF3 to ORF7 and ORF5a [24,26]. ORF1 is similarly translated into pp1a and pp1ab, which encode at least 16 nsps (nsp1*α*, nsp1*β*, nsp2, nsp2NF, nsp2TF, nsp3–6, nsp7*α*, nsp7*β* and nsp8–12). ORF2a, ORF2b, ORF3, ORF4, ORF5, ORF5a, ORF6 and ORF7 are located upstream of the 3′ untranslated region (UTR) and encode 8 structural proteins, including GP2a, 2b (E), GP3, GP4, GP5, ORF5a, M and N, respectively [27,29]. PRRSV consists of two species: *B. europensis* (PRRSV-1, formerly European genotype) and *B. americense* (PRRSV-2, formerly American genotype) [30], which share only 50–70% nucleotide sequence identity. PRRSV-1 is further classified into four subtypes (sub1–sub4) based on the ORF5 lineage classification system, while PRRSV-2 is divided into nine lineages (L1–L9) [31].

EAV and PRRSV replicate in the cytoplasm of infected cells, with the formation of double-membrane vesicles (DMVs) that are induced by certain viral non-structural proteins (nsp2/3). The viral RNA replication and transcription complex is associated with these DMVs, where viral replication and transcription occur. Similar to other members of the order *Nidovirales*, genome replication proceeds through the synthesis of continuously transcribed, full-length negative-sense RNA, while transcription occurs via synthesis of a 3′−5′ coterminal, nested set of subgenomic, negative-sense RNAs generated by discontinuous transcription. These subgenomic RNAs subsequently function as templates for the structural gene subgenomic mRNAs. Synthesis of individual subgenomic negative-sense strands is driven by transcription-regulating sequences (TRSs) in the genome [32].

Over the last decade, various methods of reverse genetics for RNA viruses have become available, such as *in vitro* ligation [33] and transformation-associated recombination using yeast artificial chromosomes [33,34]. These traditional methods frequently induce instability of full-length cDNAs due to their large size and/or problematic sequences and toxicity in prokaryotic hosts [35]. Studies overcame these disadvantages by cloning viral genomes in multiple fragments, using low-copy-number vectors or employing bacterial artificial chromosomes (BACs) using synthetic low-copy-number plasmids based on the *Escherichia coli* F-factor [36]. More recently, non-clonal reverse genetic systems have emerged, allowing *in vitro* assembly and amplification of viral cDNAs and offering alternatives to traditional cloning techniques. These methods include Gibson assembly followed by rolling circle amplification [37,38], a cloning-free and exchangeable system for virus engineering and rescue [39], and circular polymerase extension reaction (CPER). CPER is a PCR-based bacterium-free approach that enables rapid, accurate construction of recombinant viruses by assembling overlapping cDNA fragments that span the entire genome [40,42]. In the presence of a ‘linker’ fragment that connects the 5′ and 3′ UTRs of the viral genome with functional mammalian transcription initiation and termination elements, the individual genome overlapping cDNA fragments are extended in a single PCR reaction to assemble into a circularized full-length viral cDNA clone product [43]. The circularized cDNA clone is then transfected into mammalian cells, leading to the intracellular synthesis of viral genomic RNA and, ultimately, the production of infectious progeny virus [40,44]. This method has been previously used to generate recombinant viruses of other positive-stranded RNA virus families, such as flaviviruses (virulent New South Wales isolate of the Kunjin strain of West Nile virus) [44], insect-specific Casuarina virus, alphaviruses (Ross River virus), caliciviruses (murine norovirus and HuNoV [40]) and severe acute respiratory syndrome coronavirus 2 (SARS-CoV-2) [42,45]. Recently, CPER was used to generate an infectious clone of the negative-sense RNA mononegavirus, respiratory syncytial virus (RSV), by co-transfecting cells with the circular cDNA and helper plasmids encoding viral proteins essential for initiating viral replication [46].

Development of infectious cDNA clones of arteriviruses has been challenging, particularly due to their compact and overlapping gene arrangement that frequently affects their stability in bacterial hosts when cloned into plasmid vectors for reverse genetic applications. In line with this, point mutations and spontaneous deletions often occur during bacterial growth due to toxic viral sequences and cryptic transcriptional elements [7,47]. Additionally, their wide genetic diversity associated with quasi-species and, notably for PRRSV, its high recombination frequency and wide genetic heterogeneity add additional limitations for traditional reverse genetic platforms, making it difficult to keep up with the quick evolution of newly discovered, emerging field strains and our ability to study them [47]. To circumvent the above limitations associated with conventional arterivirus reverse genetic platforms, the goal of the current study was to implement CPER as a rapid, reportedly high-fidelity and versatile method for generating infectious clones of the moderately virulent equine arteritis virus KY84 strain (EAV KY84) and the porcine reproductive and respiratory syndrome virus VR2332 strain (PRRSV VR2332) (PRRSV-2 prototype), including the incorporation of reporter genes. Successful implementation of this method for members of the family *Arteriviridae* opens an important advancement in arterivirus reverse genetics, facilitating studies to understand viral gene function, viral tropism and virus–host interactions.

## Methods

### Cells and viruses

Baby hamster kidney-21 cells (BHK-21) [passage level 22 to 41, CCL-10™, American Type Culture Collection (ATCC), Manassas, VA] were maintained in Minimum Essential Medium with Earle’s salts and l-glutamine (EMEM; Corning^®^, NY, USA, Cat. No. 10–010-CV), 10% heat-inactivated fetal bovine serum (FBS, Hyclone^®^, Logan, UT, USA, Cat. No. SH30396.03), penicillin/streptomycin (10,000 U ml^−1^, 10,000 µg ml^−1^; Gibco^®^, Grand Island, NY, USA, Cat. No. 15140–122) and amphotericin B (250 µg ml^−1^, Gibco^®^, Cat. No. 15290018).

African green monkey kidney epithelial cells (MARC-145 cells, passage level 57 to 99, ATCC CRL-11171) were maintained in MEM with Earle’s salts and l-glutamine, 10% heat-inactivated FBS, 0.1 mM non-essential amino acids, 100 mM sodium pyruvate (Gibco^®^, Cat. No. 11360–070), penicillin/streptomycin and amphotericin B.

Equine pulmonary artery endothelial cells (EECs, passage level 9 to 22) were maintained in Dulbecco′s MEM (DMEM) with high glucose [4.5 g/L], L-glutamine and sodium pyruvate (Corning^®^, Cat. No. 10-013-CV), supplemented with 10% ferritin-supplemented bovine calf serum (Hyclone^®^, Cat. No. SH30072.03), 0.1 mM non-essential amino acids (Gibco^®^, Cat. No. 11140-050), penicillin/streptomycin, and amphotericin B.

EAV KY84 was propagated in EECs as described previously [48]. PRRSV VR2332 was propagated in MARC-145 cells. Virus stocks were titrated via plaque assay in all cases.

### Viral genomic RNA extraction and first-strand cDNA synthesis

Viral genomic RNA was extracted from infectious tissue culture fluids containing either EAV KY84 or PRRSV VR2332 using the taco™ DNA/RNA Extraction Kit (GeneReach, Taichung, Taiwan, Cat. No. atc-d/rna), according to the manufacturer’s instructions. Viral RNA was reverse transcribed to cDNA using the LunaScript^®^ RT SuperMix Kit (NEB, Ipswich, MA, USA, Cat. No. E3010S), which contained both oligo(dT) and random hexamer primers. The reverse transcription reaction was performed in a 20 µl reaction mixture containing 4 µl LunaScript RT SuperMix (5×), 6 µl nuclease-free water (NFW) and 10 µl template RNA. Thermal cycling was performed on a Mastercycler^®^ PCR Thermocycler (Eppendorf, Hamburg, Germany) using the following conditions: primer annealing at 25 °C for 2 min, followed by cDNA synthesis at 55 °C for 20 min and heat inactivation at 95 °C for 1 min. Following reverse transcription, an RNase treatment was performed by adding 0.4 µl of RNase H (5,000 U ml^−1^, NEB, Cat. No. M0297S), followed by an incubation at 37 °C for 20 min, before storage at −80 °C until further use.

### Designing overlapping cDNA fragments spanning the full-length EAV KY84 and PRRSV VR2332 genomes

**Fig. 1.**
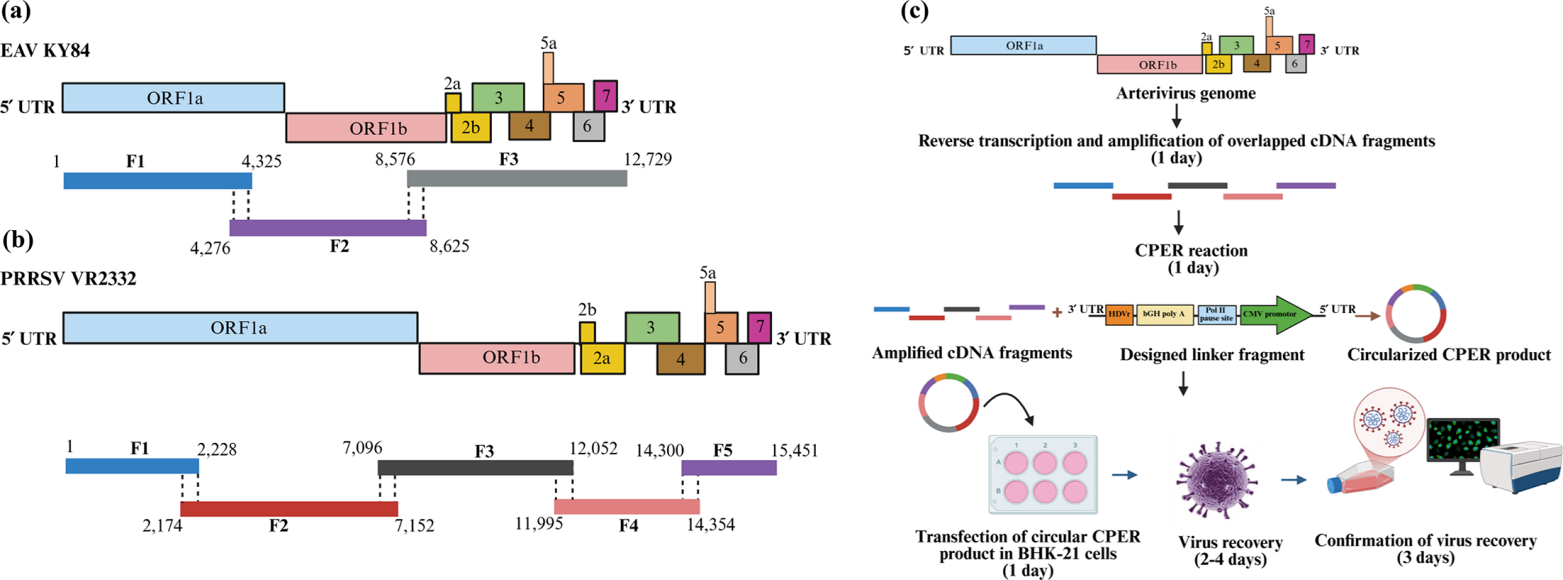
Designing of the three overlapped cDNA fragments spanning the full-length genome of EAV KY84 strain (GenBank accession number: MG137429) (F1 : 1 to 4,325; F2 : 4,276 to 8,625; and F3 : 8,576 to 12,729) (**a**). Designing of the five overlapping cDNA fragments spanning the full-length genome of PRRSV VR2332 strain (GenBank accession number: AY150564) (F1 : 1 to 2,228; F2 : 2,174 to 7,152; F3 : 7,096 to 12,052; F4: 11,995 to 14,354; and F5: 14,300 to 15,451) (**b**) to be used in the CPER assay. (**c**) Schematic representation of the reverse genetics systems based on CPER used for the generation of arterivirus infectious cDNA clones.

SnapGene software (GSL Biotech, Boston, MA) was used to design three overlapping cDNA fragments spanning the full-length genome of EAV KY84 strain (GenBank accession number: MG137429), with 50 overlapping nucleotides and a melting temperature of approximately 60 °C. The fragments (F1, F2 and F3) spanned nt 1 to 4,325 (F1), 4,276 to 8,625 (F2) and 8,576 to 12,729 (F3) (Fig. 1a). Similarly, five overlapping cDNA fragments spanning the full-length genome of PRRSV VR2332 strain (GenBank accession number: AY150564) were designed (F1 spanning nt 1 to 2,228; F2 spanning nt 2,174 to 7,152; F3 spanning nt 7,096 to 12,052; F4 spanning nt 11,995 to 14,354; and F5 spanning nt 14,300 to 15,451) with 46–55 overlapping nucleotides and a melting temperature of approximately 60 °C (Fig. 1b).

### High-fidelity PCR amplification of overlapping cDNA fragments

The designed overlapping cDNA fragments of EAV KY84 strain (F1 to F3) and PRRSV VR2332 strain (F1 to F5) were amplified using the high-fidelity Platinum^™^ SuperFi II PCR Master Mix Kit (Invitrogen^™^, Waltham, MA, USA, Cat. No. 12368010). The specific primers (Table 1) were designed using SnapGene software and synthesized by Eurofins Genomics (Louisville, KY, USA). A total reaction volume of 50 µl was prepared, containing 25 µl 2× Platinum^™^ SuperFi II PCR Master Mix, 2.5 µl of forward primer (10 µM), 2.5 µl of reverse primer (10 µM), 15 µl of NFW and 5 µl of cDNA template. Thermal cycling was performed on a Mastercycler^®^ ×50 PCR Thermocycler using the following conditions: an initial denaturation step at 98 °C for 30 s, followed by 30 cycles of denaturation at 98 °C for 10 s, annealing at 60 °C for 10 s, and extension at 72 °C for 3 min. Then, a final extension at 72 °C for 5 min.

**Table 1.** Primer sequences used to amplify the overlapping cDNA fragments of EAV KY84 strain (GenBank accession number: MG137429) and PRRSV VR2332 strain (GenBank accession number: AY150564) genomes and linker-specific sequences

Virus (strain)	Primer name	**Primer sequence (5′ to 3**′)	bp	GC %	Tm °C	Nucleotide position (5′ to 3′)
**EAV** (**KY84**)	EAV-F1-FP	***aagcagagctcgtttagtgaaccgt***GCTCGAGGTGTGTATGGTGCCATATACGG	54	51.8	76.9	1–29
	EAV-F1-RP	TCCAAACCACATTTCAGAGAGGTACGACGTGCCAGCAGCAGCAATATAAAACCAC	55	47.3	75.2	4,325–4,271
	EAV-F2-FP	TTTATATTGCTGCTGCTGGCACGTCGTACCTCTCTGAAATGTGGTTTGGAGGCTATCCC	59	49.2	76.6	4,276–4,334
	EAV-F2-RP	CCCTCATTAGACCAAAGGTCAATTTCTTGGCCCAGATGACATGCTATAGGTGGG	54	48.2	75.4	8,625–8,572
	EAV-F3-FP	CCTATAGCATGTCATCTGGGCCAAGAAATTGACCTTTGGTCTAATGAGGGCCTCGAG	57	49.1	76.3	8,576–8,632
	EAV-F3-RP	*ttttttttttttttttttttttttt*GGTTCCTGGGTGGCTAATAACTACTTCAACATGACG	61	27.9	68.1	12,704–12,669
	EAV-F4_Linker-FP	AGTAGTTATTAGCCACCCAGGAACC*aaaaaaaaaaaaaaaaaaaaaaaaaaaaaaaaaggccggcatggtcccag*	75	33.3	71.9	12,680–12,704
	EAV-F4_Linker-RP	ATATGGCACCATACACACCTCGAGC*acggttcactaaacgagctctgcttatatagacct*	60	46.7	75.7	25–1
**PRRSV (VR2332**)	PRRSV-F1-FP	*aagcagagctcgtttagtgaaccgt*TATGACGTATAGGTGTTGGCTCTATGC	52	46.2	75.7	1–27
	PRRSV-F1-RP	CCTCAACCCCAGGGAGCACAACATCCCAATCAAAGGAGGTGTCGATTACGCGTGG	55	56.4	78.9	2,228–2,174
	PRRSV-F2-FP	CCACGCGTAATCGACACCTCCTTTGATTGGGATGTTGTGCTCCCTGGGGT	50	56	77.9	2,174–2,223
	PRRSV-F2-RP	ATTTTGGACCCAGCAAGGACTCTGGTCTCAATGGCTTGGAGGGTATGCTTGGTGC	55	52.7	77.4	7,152–7,098
	PRRSV-F3-FP	TAGCACCAAGCATACCCTCCAAGCCATTGAGACCAGAGTCCTTGCTGGGTCCAA	54	53.7	77.7	7,096–7,149
	PRRSV-F3-RP	GTTCAATGACAGGGCCCGGGGGAAAATAAAACTTCAAGCTGGTGGCAGTGGC	52	53.8	77.4	12,052–12,001
	PRRSV-F4-FP	TATAAGGCCACTGCCACCAGCTTGAAGTTTTATTTTCCCCCGGGCCCTGTCATTGA	56	50	76.5	11,995–12,050
	PRRSV-F4-RP	ATAGGGGTTGCCACGGAACCATCAAGCACAACTCTTTTGAGGTCGATCAGATGACCTTCGAC	62	50	77.3	14,354–14,293
	PRRSV-F5-FP	GTCATCTGATCGACCTCAAAAGAGTTGTGCTTGATGGTTCCGTGGCAACCCC	52	51.9	76.6	14,300–14,351
	PRRSV-F5-RP	TTTTTTTTTTTTTTTTTTTTTTTTTTTTTTTTAATTTCGGCCGCATGGTTCTCGC	55	23.6	65.5	15,444–15,390
	PRRSV-F6_Linker-FP	TGGCGAGAACCATGCGGCCGAAATTAAAAAAAAAAAAAAAAAAAAAAAAAAAAAAAAAAAAAAA*ggccggc*	71	29.6	70	15,388–15,451
	PRRSV-F6_Linker-RP	CTCCTGACAATACAAATGCCAAGGCATAGAGCCAACACCTATACGTCATA*acggttcactaaacgagctct*	71	45.1	76.3	50–1

Italic, underlined and lower-case nucleotide sequences indicate the linker sequence.

Tm, melting temperature.

The amplified PCR products were analysed by gel electrophoresis on a 1% agarose gel, visualized with a Dark Reader Transilluminator (Clare Chemical Research, DR-89X, Dolgeville, New York, USA), excised and purified using the Monarch DNA Gel Extraction Kit (NEB, Cat. No. R045A) according to the manufacturer’s instructions. PCR products were eluted in 10 µl of NFW and quantified using the Qubit^™^ dsDNA BR Assay Kit (Invitrogen^™^, Cat. No. Q33231) on a Qubit^™^ 4 Fluorometer (Invitrogen^™^, Cat. No. 2332624120169).

### Linker sequence design and amplification

Specific linker sequences for EAV (pEAV-linker) and PRRSV (pPRRSV-linker) were designed and synthesized as described by Liu and Gack [43]. The pEAV-linker (1,125 bp) was designed to encode the following (5′ to 3′): the last 25 nt of the 3′ UTR (5′ AGTAGTTATTAGCCACCCAGGAACC 3′) and the poly(A)_33_ tail of the EAV KY84 strain genome (GenBank accession number: MG137429), the hepatitis delta virus ribozyme (HDVr), the bovine growth hormone (BGH) poly (A) signal, the RNA polymerase II (Pol II) transcriptional pause signal, the cytomegalovirus (CMV) promoter and the first 25 nucleotides of the viral 5′ UTR (5′ GCTCGAGGTGTGTATGGTGCCATAT 3′) of EAV KY84 strain (GenBank accession number: MG137429). Similarly, the pPRRSV-linker (1,138 bp) encoded the sequences described above flanked by the last 32 nt of the 3′ UTR (5′ ATTTAATTGGCGAGAACCATGCGGCCGAAATT 3′), poly(A)_39_ tail and the first 25 nt of the viral 5′ UTR (5′ TATGACGTATAGGTGTTGGCTCTAT 3′) of PRRSV VR2332 strain (GenBank accession number: AY150564). Both linker sequences were synthesized and cloned into the pUC19 vector by a commercial company (GeneArt, Regensburg, Germany).

DNA was extracted from both linker plasmids using the Nucleospin plasmid transfection-grade DNA purification kit (Takara Bio, San Jose, CA, USA, Cat. No. 740490.50), and the concentration was measured using the Qubit^™^ dsDNA BR Assay Kit. The linker sequences were amplified using specific primers (Table 1) as described above, with 5 µl of linker-purified DNA at 10 ng µl^−1^ as the template. Amplified linker fragments were similarly analysed by gel electrophoresis, and PCR products were extracted and quantified as indicated above.

### CPER reaction, transfection of permissive cells and virus recovery

The CPER reaction was performed on a 50 µl reaction volume using the high-fidelity PrimeSTAR Max DNA polymerase (Takara Bio, Cat. No. R045A). The reaction included equimolar amounts (0.05 or 0.1 pmol) of each purified cDNA fragment of EAV KY84 (F1 to F3 and the pEAV-linker) or PRRSV VR2332 (F1 to F5 and the pPRRSV-linker), 10 µl of PrimeSTAR GXL buffer 5×, 4 µl of dNTP mixture and 2 µl of PrimeSTAR GXL DNA polymerase, and the volume was completed with NFW to reach 50 µl. The cycling conditions of the CPER reaction were as follows: an initial denaturation at 98 °C for 2 min, 35 cycles of denaturation at 98 °C for 10 s, annealing at 55 °C for 15 s, extension at 68 °C for 15 min and final extension at 68 °C for 15 min (reaction time was ~9.5 h). Reaction products were kept at room temperature (RT) until transfection to avoid DNA precipitation. Negative controls for the CPER reaction were prepared by using cDNA fragments, excluding F1/F3 (minus F1/F3) of EAV KY84 and F2/F3 (minus F2/F3) of PRRSV VR2332.

For cell transfection, a total of 4.5×10^5^ BHK-21 cells in 2.5 ml complete EMEM per well were seeded in 6-well plates and incubated in a cell culture incubator at 37 °C and 5% CO_2_ for 24 h. The following day, the transfection reaction was prepared as follows: 225 µL of Opti-MEM^®^I (1 x) Reduced Serum Medium (Gibco^®^, Cat. No. 31985–062) pre-warmed at 37 °C, 25 µl of the final CPER product without purification (0.05 and 0.1 pmol DNA) and 6 µl of *TransIT-X2* Dynamic delivery system (Mirus Bio, Madison, WI, USA, Cat. No. MIR 6003) equilibrated to RT. The complexes were incubated at RT for 30 min. During this time, the existing culture medium was carefully removed from each well, the cells were gently rinsed twice with 2.5 µl of EMEM media with 10% FBS and the cells were overlaid with 2.5 ml of the same media. After the incubation time, the transfection mixture was added dropwise to different areas of the wells in a slow manner, and the plates were gently rocked and incubated at 37 °C and 5% CO_2_. At 24 h post-transfection (hpt), the culture media was replaced with EMEM containing 2% FBS and penicillin/streptomycin, and the plates were incubated until 72 hpt (for PRRSV VR2332) or until cytopathic effect (CPE) became visible (EAV KY84). Mock and *TransIT-X2* negative controls were included in each transfection experiment. At 72 hpt, the plates were subjected to three rounds of freeze-thawing at −80 °C, the cell lysates were clarified by centrifugation (500 ***g*** for 10 min at 4 °C), labelled as recombinant EAV KY84 (rEAV KY84) and recombinant PRRSV VR2332 (rPRRSV VR2332) passage 0 (P0) and stored at −80 °C for further use. The entire CPER assay is summarized in Fig. 1c.

### Propagation of recovered EAV KY84 (rEAV KY84) and PRRSV VR2332 (rPRRSV VR2332)

A total of 5.5×10^5^ of BHK-21 cells and 6.5×10^5^ MARC-145 cells were seeded in 6-well plates and incubated at 37 °C and 5% CO_2_ for 24 h to be used for the propagation of rEAV KY84 and rPRRSV VR2332, respectively. The next day, a 1 : 2 dilution of rEAV KY84 (P0) and rPRRSV VR2332 (P0) was prepared in EMEM containing 2% FBS, and 500 µl of each was inoculated into the seeded BHK-21 and MARC-145 monolayers, respectively. After 1 h of adsorption at 37 °C, 2 ml of EMEM containing 2% FBS was added to each well, and the plates were incubated until CPE was observed. CPE on BHK-21 induced by rEAV KY84 (P1) was noted after 48–72 hours post-infection (hpi), and CPE on MARC-145 induced by rPRRSV VR2332 (P1) became evident between 72 and 96 hpi. Negative controls (Mock, *TransIT-X2* and minus F1/F3 of EAV KY84 or minus F2/F3 of PRRSV VR2332) were also included in these experiments.

### Immunofluorescence

A total of 7.5×10^4^ BHK-21 cells were seeded in 24-well plates containing 12-mm circular micro cover glass (VWR, Radnor, PA, Cat. No. 89015-725). The next day, rEAV KY84 P0 was diluted 1 : 4 in EMEM containing 2% FBS, and 200 µl were inoculated and adsorbed for 1 h at 37 °C in a humidified 5% CO_2_. An additional 300 µl of media was added to each well, and the plates were incubated until ~20% CPE was observed (usually 24 hpi). Negative controls (Mock, *TransIT-X2* and minus F1/F3) were included. At ~24 hpi, infected cells were fixed with 500 µl of 4% paraformaldehyde (Sigma-Aldrich, St. Louis, MO, USA, Cat. No. P6148-500G) in 1× PBS (pH=7.4) for 1 h at RT. Following 3× washes with 1× PBS/10 mM glycine (Alfa Aesar, Tewksbury, MA, USA, Cat. No. A13816), cells were permeabilized by adding 500 µl of 0.1% Triton X-100 (Sigma-Aldrich, St. Louis, MO, USA, Cat. No. 1001748095×100–500 ml) for 15 min at RT. The cells were washed three times, and 500 µl of blocking solution (5% fetal bovine serum in 1×PBS) were added and incubated at RT for 30 min. Subsequently, the cells were incubated with 200 µl of anti-nucleocapsid (N; MAb 3E2) [49] or anti-nonstructural protein 1 (nsp1; MAb 12A4) [2] monoclonal antibodies diluted 1 : 100 and 1 : 800 in blocking solution, respectively. Following a 1-h incubation at RT, the monolayers were washed 3×, 200 µl of a F(ab′)2-Goat anti-Mouse IgG (H+L) Cross-Adsorbed Secondary Antibody, Alexa Fluor^™^ 488 (Invitrogen, Cat. No. A-11017) (diluted at 1 µg ml^−1^) (1 : 200) in blocking solution was added to each well and the cells were incubated for 45 min at RT in the dark. Finally, 22 µl of Hoechst 33342 solution (20 µg ml^−1^, Invitrogen, Cat. No. H3570) was added to each well, and the cells were incubated for an additional 15 min. After washing, the coverslip was mounted on a glass slide using Fluomount-G^™^ mounting medium (SouthernBiotech, Birmingham, AL, USA, Cat. No. 0100-01). All coverslips were observed using a Nikon Eclipse Ti2 fluorescence microscope (Nikon, NY, USA).

For rPRRSV VR2332, a total of 1×10^5^ MARC-145 cells were seeded in 24-well plates containing cover glasses and, after 24 h, were infected with 200 µl of a 1 : 2 dilution of rPRRSV VR2332 P0. The cells were incubated until ~20% CPE was obtained (usually 48 hpi). The infected cells were then fixed, permeabilized and blocked as indicated above. Cells were incubated in 200 µl of rabbit anti-GP5 anti-peptide antisera (OBS, 182 : 200, rabbit no PA6501, developed by Dr Udeni Balasuriya) and mouse anti-nucleocapsid (N) monoclonal antibody (SDOW-17-A, Rti LLC, Brookings, SD, USA), both diluted to 1 : 100 in blocking buffer. Then, F(ab′)2-Goat anti-Rabbit IgG (H+L) Cross-Adsorbed Secondary Antibody, Alexa Fluor^™^ 488 (Invitrogen, Cat. No. A-11070) or F(ab′)2-Goat anti-Mouse IgG (H+L) Cross-Adsorbed Secondary Antibody, Alexa Fluor^™^ 488 (Invitrogen, Cat. No. A-11017) diluted at 1 µg ml^−1^ (1 : 200) in blocking solution was used as secondary antibodies, respectively. Stained cover glasses were mounted and visualized as indicated above.

### Development of EAV KY84 and PRRSV VR2332 reporter viruses using CPER

**Fig. 2.**
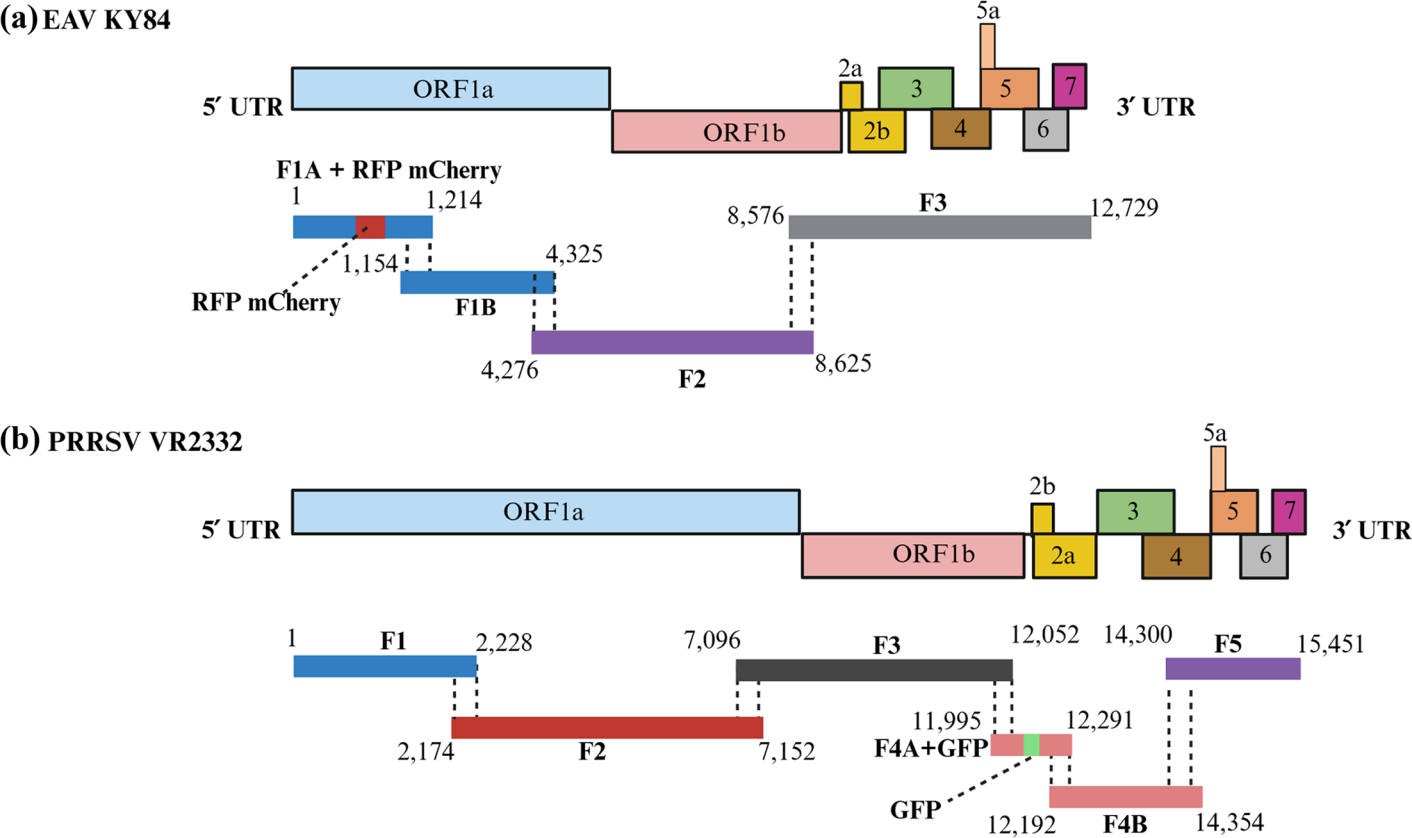
Designing of the overlapped cDNA fragments spanning the full-length genome incorporating reporter genes of EAV KY84-RFP mCherry, in which the RFP mCherry was inserted into ORF1a at a position located five codons downstream of the nsp1/nsp2 cleavage junction (**a**) and PRRSV VR2332-GFP, in which the GFP was inserted between the stop codon of ORF1b and the start codon of ORF2a (**b**) for use in the CPER assay.

The previously designed EAV KY84 F1 (1 to 4,325 bp), which spans ORF1a, was subdivided into two overlapping fragments, F1A (1 to 1,214 bp) and F1B (1,154 to 4,325 bp), using SnapGene software. Then, red fluorescent protein (RFP) mCherry reporter gene (792 bp) was inserted into F1A following the previously reported strategy used with the virulent Bucyrus strain of EAV [50] (Fig. 2a). Briefly, a DNA linker (69 nt) encoding the 23-amino-acid FMDV 2A oligopeptide was inserted in frame at the 3′ end of the mCherry coding sequence (708 nt encoding the 236-aa RFP) derived from the pME-mCherry-T2A-EGFP vector (GenBank accession number JN717245). This mCherry-FMDV2A was inserted into ORF1a at the position located five codons downstream of the nsp1/nsp2 cleavage junction. Thus, the first five codons of nsp2 were maintained to ensure processing of the nsp1/mCherry junction by the nsp1 protease. The first amino acid of nsp2 (Gly) was replaced by Pro to promote 2A activity. The codon usage for nsp2 residues 2–5 was altered (GGCTACAATCCACCC to CCCTATAACCCGCCA) to minimize the chance of DNA/RNA recombination with the corresponding sequence directly upstream of the mCherry-2A cassette. The designed F1A with the inserted RFP mCherry (F1A-RFP mCherry) was synthesized and cloned into the pUC19 vector by a commercial company (GeneArt), and the construct was named pEAVKY84-Frag1A-RFP mCherry.

Likewise, the designed PRRSV VR2332 F4 (11,995 to 14,354 bp) was subdivided into two fragments, F4A (11,995 to 12,291 bp) and F4B (12,191 to 14,354 bp) (Fig. 2b), as an initial step for insertion of the GFP into F4A. Nucleotide sequences encoding GFP were inserted into fragment F4A as illustrated before by Pei *et al*. [51], who described the strategy for inserting the GFP gene in the North American PRRSV strain P129. Briefly, the GFP gene sequence utilized by Pei *et al*. and reportedly derived from the pEGFP-N1 plasmid (Clontech, Takara) was inserted between the stop codon of ORF1b and the start codon of ORF2a. This non-coding region is comprised of only one adenosine nucleotide. The TRS associated with ORF2a and ORF2b (TRS2; TGAACC) is positioned 26 nt upstream from the start of ORF2a and is embedded in ORF1b. Upon GFP insertion into the region, TRS2 will drive transcription of the GFP gene rather than ORF2a and ORF2b. Thus, a synthetic TRS (TTAACC) with flanking sequences derived from TRS6 was introduced 22 nt downstream of GFP and 17 nt upstream of the ORF2a start. F4A-GFP was synthesized and cloned into the pUC19 vector by a commercial company (GeneArt), and the construct was named pPRRSV VR2332-Frag4A-GFP.

Subsequently, the F1A-RFP mCherry of pEAVKY84-Frag1A-RFP mCherry, the F1B of EAV KY84, the F4A-GFP of pPRRSV VR2332-Frag4A-GFP and the F4B of PRRSV VR2332 were amplified using a specific set of primers (Table 2) and included in their corresponding CPER reactions with other fragments as indicated previously. BHK-21 cells were seeded and transfected with the CPER products as indicated above. RFP mCherry and GFP signals in transfected BHK-21 were visualized directly using Incucyte^®^ S3 Live-Cell Analysis System (Sartorius, Göttingen, Germany) every 24 h.

**Table 2.** Primer sequences used for amplification of F1A-RFP mCherry and F1B of EAV KY84 and F4A-GFP and F4B of PRRSV VR2332

Virus (strain)	Primer name	Primer sequence (5′ to 3′)	bp no	Gc %	Tm °C	Nucleotide position (5′ to 3′)
**EAV KY84**	EAV-F1A -FP	*aagcagagctcgtttagtgaaccgt*GCTCGAGGTGTGTATGGTGCCATATACGG	54	51.8	76.9	1 to 29
	EAV-F1A- RP	CTTGTCACAAATCATCACGTGCTTGGCGTTCGGACAAACACGCCCACCTGGGA	53	54.7	77.9	1,214–1162
	EAV-F1B- FP	GGTTACCATCCCAGGTGGGCGTGTTTGTCCGAACGCCAAGCACGTGATGATTTGTGACAAG	61	54.1	78.9	1,154–1214
	EAV-F1B- RP	TCCAAACCACATTTCAGAGAGGTACGACGTGCCAGCAGCAGCAATATAAAACCAC	55	47.3	75.2	4,325–4271
**PRRSV VR2332**	PRRSV-F4A- FP	TATAAGGCCACTGCCACCAGCTTGAAGTTTTATTTTCCCCCGGGCCCTGTCATTGA	56	50	76.5	11,995–12,050
	PRRSV-F4A- RP	CTTCTGTAATTGCTCAGAGTGAATGGCAGGGCGCGTACGGAGTATCGCGGAGCAAACCAATCTGATGCAAAAGACCACCAG	81	51.8	80	12,291–12,211
	PRRSV-F4B- FP	CTTCACCATCGCCGGTTGGCTGGTGGTCTTTTGCATCAGATTGGTTTGCTCCGCGATACTCCGTACGCGC	70	57.1	81.2	12,192–12,261
	PRRSV-F4B- RP	ATAGGGGTTGCCACGGAACCATCAAGCACAACTCTTTTGAGGTCGATCAGATGACCTTCGAC	62	50	77.3	14,354–14,293

Italic, underlined and lower-case nucleotide sequences indicate the linker sequence.

Tm, melting temperature.

Recovery of the rEAV-RFP mCherry and rPRRSV VR2332-GFP following one passage was confirmed by immunofluorescence as described above, but utilizing F(ab′)2-Goat anti-Rabbit IgG (H+L) Cross-Adsorbed Secondary Antibody, Alexa Fluor^™^ 594 (Invitrogen, A-11012), and F(ab′)2-Goat anti-Mouse IgG (H+L) Cross-Adsorbed Secondary Antibody, Alexa Fluor^™^ 594 (Invitrogen, Cat. No. A-11020), for rPRRSV VR2332-GFP as appropriate.

### Next-generation sequencing and bioinformatics

The viral RNA of the parental and CPER-generated viruses with or without reporter genes of EAV KY84 and PRRSV VR2332 (P2) were treated with TURBO DNase (Invitrogen, Cat. No. AM2239) at 37 °C for 30 min and then extracted using MagMAX^™^ Pathogen RNA/DNA Kit (Thermo Fisher Scientific, Cat. No. 4462359) with KingFisher^™^ Flex System (Thermo Fisher Scientific, Cat. No. 5400630) following the manufacturer’s guidelines [52]. Double-stranded cDNA was synthesized using the UltraClean ds-cDNA Synthesis Module (Vazyme, Nanjing, China, Cat. No. UNR201). cDNA was bead-purified using AMPure XP Beads (Beckman Coulter, Brea, CA, USA, Cat. No. A63881), and DNA concentration was measured with the Qubit 1× dsDNA High Sensitivity Kit (Thermo Fisher Scientific, Cat. No. Q33231) on a Qubit Flex Fluorometer (Thermo Fisher Scientific, Cat. No. Q32866). DNA libraries were prepared using Illumina DNA Prep library preparation kit (96 samples) (Illumina, Cat. No. 20060059) and Illumina DNA/RNA UD Index (96 Indexes, 96 Samples) (Illumina, Cat. No. 20091654). The library pool was sequenced following standard Illumina protocols at the next-generation sequencing (NGS) section in the Iowa State University-Veterinary Diagnostic Laboratory, on an Illumina MiSeq i100 Plus platform (Illumina, Cat. No. 20115695) with the MiSeq i100 Series 25M Reagent Kit (600 cycle) (Illumina, Cat. No. 20126568) to generate 300-bp paired-end reads. Raw reads for each sample were automatically base-called and demultiplexed on the sequencing platform using the default settings.

The raw reads per sample were quality-checked with FastQC (v0.11.9) and processed with Trimmomatic (v0.39). High-quality reads were aligned to reference sequences for EAV (or PRRSV) obtained from the NCBI database using BWA-MEM (v 0.7.17) [53]. The reads mapped to the reference sequences (MG137429, MT796267, EF532801, DQ988080, LC557039 and AY588319) were extracted using SAMtools (v1.7) and *de novo* assembled using ABySS (v2.2.4) and SPAdes (v3.13.0). The final assemblies were checked using blastn and manually curated in IGV (v2.16.0) to get a consensus sequence for the genome sequence [54]. Full-length genome sequences generated in this study were deposited in GenBank under accession numbers: PX841640 for rEAV KY84, PX849429 for rEAV KY84-RFP mCherry, PX849432 for parental PRRSV VR2332, PX849431 for rPRRSV VR2332 and PX849430 for rPRRSV VR2332-GFP. The full-length genome sequence of the parental EAV KY84 had been previously submitted to GenBank (MG137429) as part of a prior study from the authors [55].

### *In vitro* growth kinetics and titration of CPER-generated rEAV KY84 and rPRRSV VR2332 and their reporter viruses

Parental and CPER-generated EAV KY84 and PRRSV VR2332 were concentrated by ultracentrifugation as previously described [56] and titrated using plaque assay. A total of 1.5×10^4^ EECs or MARC-145 cells resuspended in 200 µl were seeded in 96-well plates, respectively. The next day, the media were removed, and cell monolayers were inoculated in triplicate with 50 µl of each CPER-generated virus and its corresponding parental strain at a multiplicity of infection (MOI) of 5. After 1 h of adsorption at 37 °C and 5% CO_2_, the inoculum was removed, the cells were washed three times with 200 µl media and 200 µl of each fresh media was added to each well. The plates were incubated at 37 °C and 5% CO_2_. Cell lysates were collected at the following time points: 0, 6, 12, 18, 24, 30, 36 and 48 for EAV KY84 parental and related CPER-generated strains and extended to 72 and 96 hpi for PRRSV VR2332 parental and CPER-generated strains. The infectious titres were then determined by the 50% tissue culture infective dose (TCID_50_). For titration using the TCID_50_ assay, supernatant from each time point was diluted ten-fold in triplicate and added to EEC or MARC-145 cells. Plates were incubated at 37 °C and 5% CO_2_ for 96 h. At 96 hpi, the CPE was recorded, and subsequently the plates were stained with a crystal violet solution (0.2%) for 1 h, washed with tap water and allowed to dry overnight. Titres were calculated using the Reed and Muench method.

### Stability of rEAV KY84-RFP mCherry and rPRRSV-GFP reporter viruses

rEAV KY84-RFP mCherry and rPRRSV-GFP were blind passaged for 15 serial passages in EECs or MARC-145 cells previously seeded in 6-well plates, respectively, and the expression of RFP mCherry and GFP was monitored in each passage using Incucyte^®^ S3 live-cell analysis system to assess their genetic stability. Expression levels were assessed using the Incucyte basic analyser software. Fluorescent signal was quantified by calculating the percentage of positive signal area within confluent cell cultures, employing a uniform thresholding algorithm across biological replicates (*n*=6). Due to the interruption in the expression of rEAV KY84-RFP mCherry at P15, RNA was extracted from the culture supernatant of infected EECs from representative passages (P2, P4, P14 and P15) using a taco^™^ DNA/RNA Extraction Kit according to the manufacturer’s instructions. Then, the extracted RNA was reverse transcribed to cDNA, and F1A-RFP mCherry was amplified as indicated above and analysed by gel electrophoresis on a 1% agarose gel.

### Statistical analysis

Statistical analysis for growth kinetics was performed using a two-way ANOVA test with Tukey’s multiple comparisons test to compare treatments at each time point. Statistical analysis for the stability of reporter viruses was performed using the Kruskal–Wallis test with Dunn’s post hoc test to compare all the serial passages to the control group (P2). Both analyses were performed using GraphPad Prism 9 (San Diego, CA, USA). Significance was set at *P*-value <0.05 in all cases.

## Results

### PCR amplification and CPER reaction for assembly of EAV KY84 and PRRSV VR2332 full-length genomes

The CPER method relies on overlap extension PCR, which combines multiple dsDNA fragments with homologous ends into a single circular product. In the current study, we successfully amplified the three overlapping cDNA fragments spanning the full-length genome of the EAV KY84 strain using a high-fidelity DNA polymerase (Fig. S1A, available in the online Supplementary Material). Similarly, the full-length PRRSV VR2332 genome was amplified in five overlapping cDNA fragments (Fig. S1B). CPER for annealing the PCR products with the specific linker sequence yielded a high-molecular-weight product (Fig. S1C, D) corresponding to the genome length, including the linker sequence.

### Rescue of infectious rEAV KY84 and rPRRSV VR2332

**Fig. 3.**
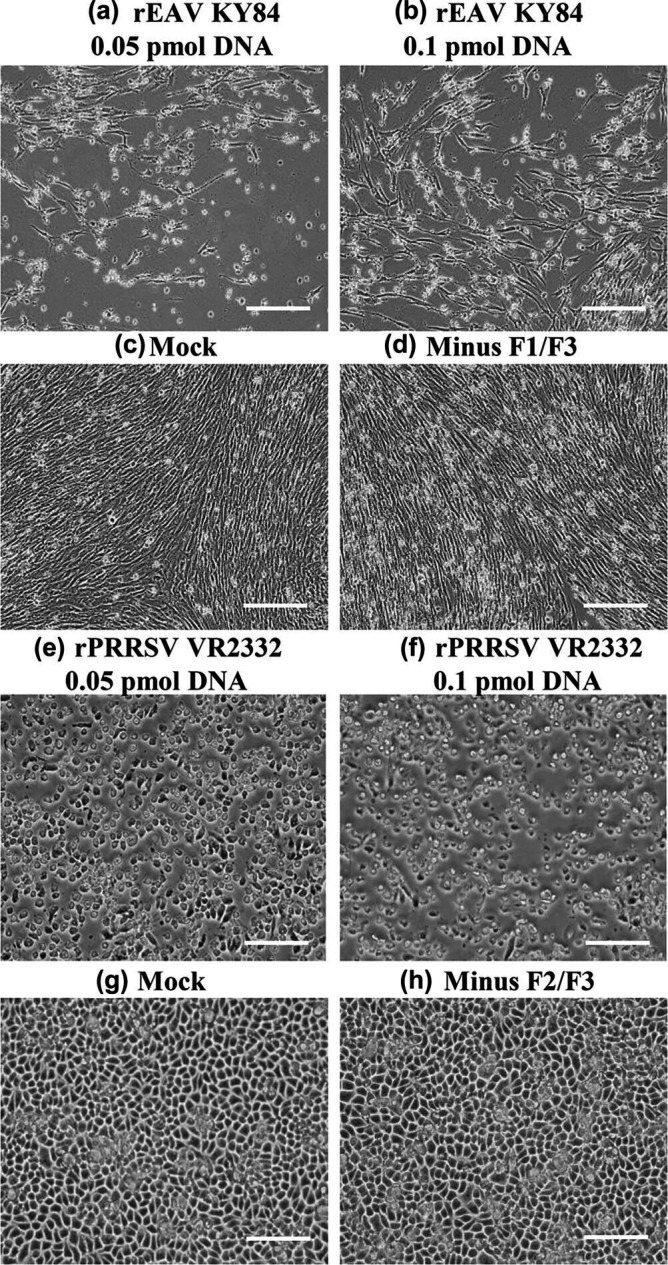
Recovery of rEAV KY84 (0.05 (a) and 0.1 (b) pmol of CPER DNA) P1 in BHK-21 cells at 48 hpi compared to the negative controls (mock-infected, non-transfected BHK-21 cells (**c**) and CPER reaction product lacking F1/F3 (**d**). Recovery of rPRRSV VR2332 (0.05 (e) and 0.1 (f) pmol of CPER DNA) P1 in MARC-145 cells at 96 hpi compared to the negative controls (mock-infected, non-transfected MARC-145 cells (**g**) and CPER reaction product lacking F2/3 (**h**)). Magnification: 20×; scale=100 µm.

BHK-21 cells are a common, easily transfectable cell line that has been previously used for recovery of infectious cDNA clones of arteriviruses (EAV and PRRSV). BHK-21 cells are both susceptible and permissive to EAV [57]. However, this cell line is permissive but not susceptible to PRRSV infection; hence, they provide high transfection efficiency with robust progeny output that can be subsequently amplified in susceptible and permissive MARC-145 cells [47,58,61]. Infectious rEAV KY84 strain was successfully rescued following transfection of BHK-21 with 0.05 and 0.1 pmol DNA of the circular CPER products of EAV KY84 strain, with specific CPE observed at 48 hpt at both DNA concentrations, and complete CPE observed at 72 hpt. Rescued rEAV KY84 (P0) was passaged once onto fresh BHK-21 cells with 80–90% CPE observed at 48 hpi (Fig. 3a–d). For rPRRSV VR2332, CPE was not observed following transfection of BHK-21 cells at P0 for either of the two DNA concentrations tested (0.05 and 0.1 pmol DNA of the circular CPER products of PRRSV VR2332 strain) until 72 hpt. At 72 hpt, the clarified supernatant derived from P0 was passaged into MARC-145 cells, and CPE was evident after 96 hpi (Fig. 3e–h).

**Fig. 4.**
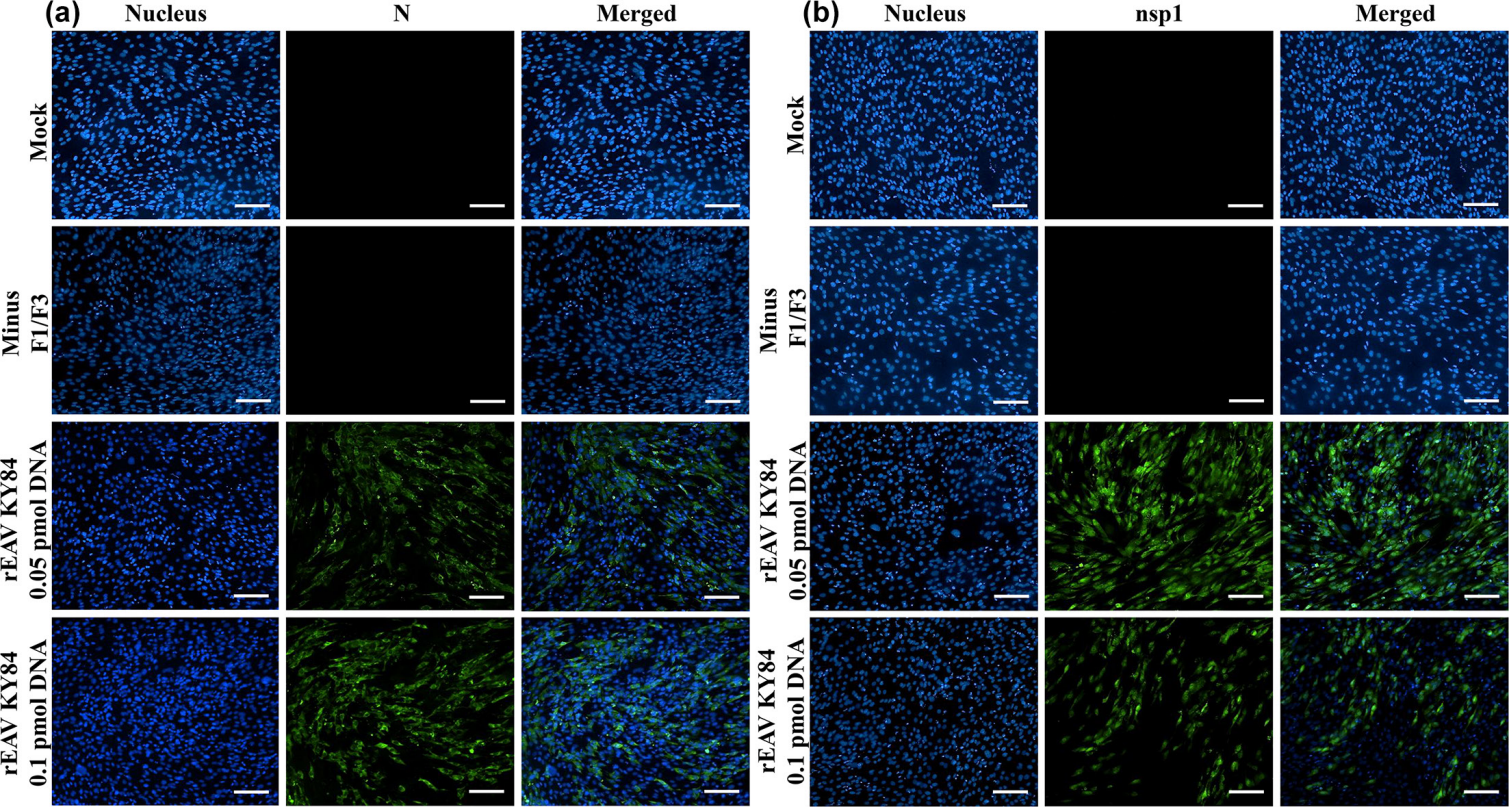
Confirmation of rEAV KY84 recovery following CPER. BHK-21 cells were infected with rEAV KY84 P0 (recovered from 0.05 and 0.1 pmol of CPER DNA) and immunolabelled with anti-N (**a**) and anti-nsp1 (**b**) antibodies at 24 hpi. Mock-infected and minus F1/F3 were included as negative controls. Magnification: 20×; scale: 100 µm.

**Fig. 5.**
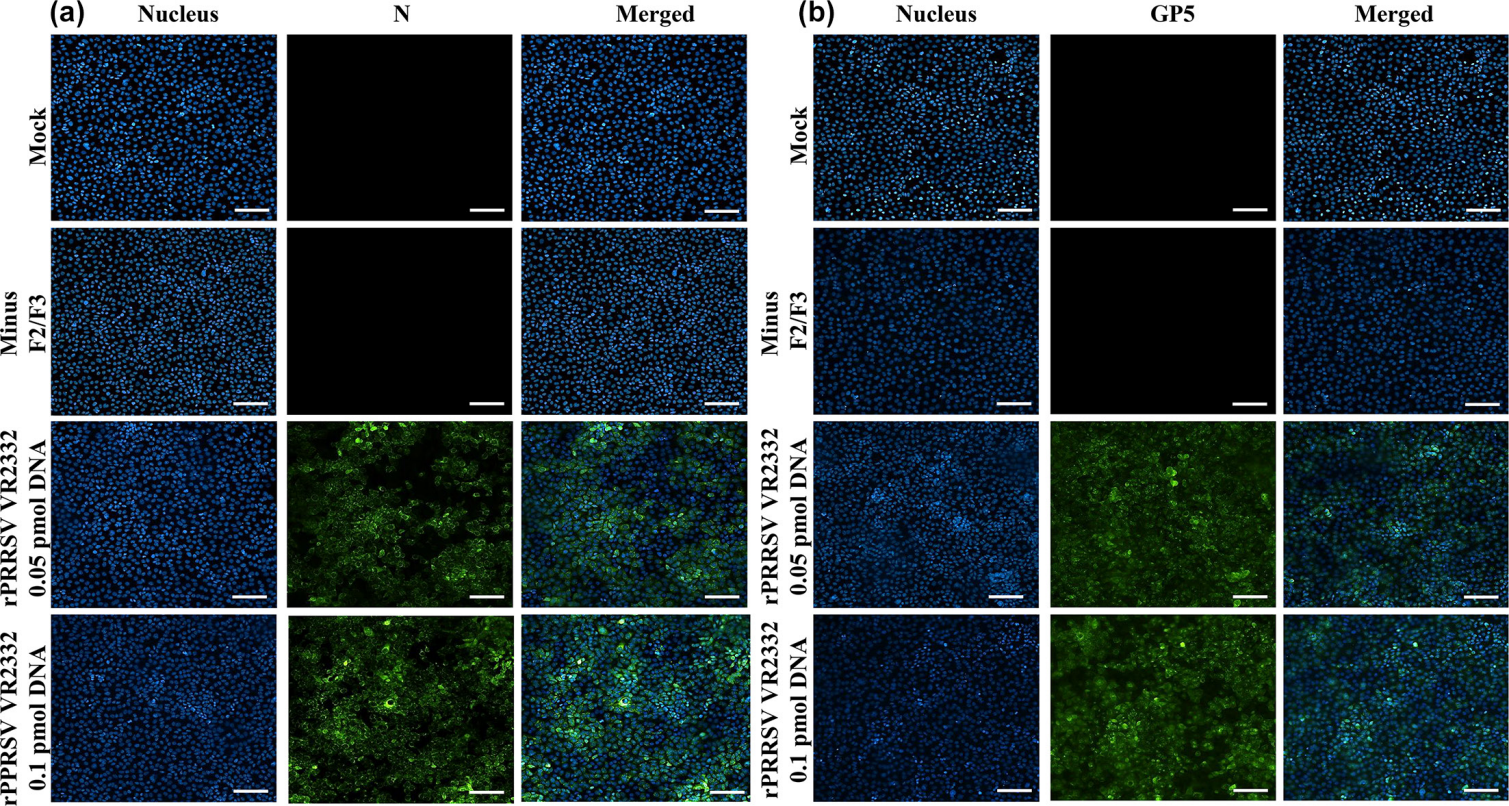
Confirmation of rPRRSV VR2332 recovery following CPER. MARC-145 cells were infected with rPRRSV VR2332 P0 (recovered from 0.05 and 0.1 pmol of CPER DNA) and immunolabelled with anti-N (**a**) and anti-GP5 (**b**) antibodies at 48 hpi. Mock-infected and minus F2/F3 were included as negative controls. Magnification: 20×; scale: 100 µm.

To confirm recovery of infectious progeny virus from BHK-21 cells transfected with assembled cDNA clones of EAV KY84 and PRRSV VR2332, the tissue culture fluids from P0 were inoculated (~MOI of 1) into BHK-21 or MARC-145 cells and stained with viral protein-specific monoclonal antibodies or antiserum at 24 or 48 hpi, at which time there was evidence of CPE for rEAV KY84 and rPRRSV VR2332, respectively. BHK-21 cells infected with rEAV KY84 were strongly immunolabelled with an anti-N as well as an anti-nsp1 monoclonal antibody (Fig. 4a, b). Similarly, rPRRSV VR2332-infected MARC-145 cells were strongly immunolabelled with an anti-GP5 rabbit polyclonal and an anti-N mouse monoclonal antibody (Fig. 5a, b). These findings demonstrated successful recovery of rEAV KY84 and rPRRSV VR2332 using the standardized CPER protocol employed in the current study.

### Generation of rEAV KY84-RFP mCherry and rPRRSV VR2332-GFP reporter viruses via CPER

**Fig. 6.**
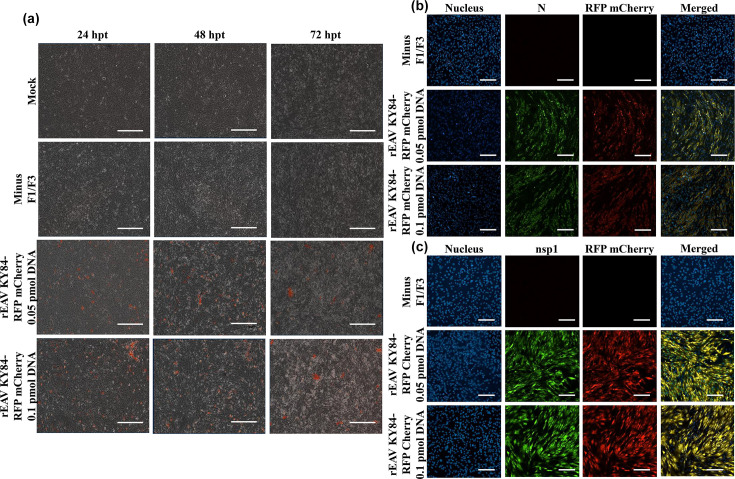
Live fluorescence imaging monitoring expression of RFP mCherry reporter gene in rEAV KY84-RFP mCherry P0 in transfected BHK-21 from 24 to 72 hpt (**a**); magnification: 4×; scale: 400 µm. Recovery of the reporter virus was confirmed by immunofluorescence using anti-N (**b**) and anti-nsp1 antibodies (**c**). Magnification: 20×; scale: 100 µm.

To demonstrate that CPER is a versatile reverse genetics tool for generating reporter arteriviruses, we used the technique to generate rEAV KY84 and rPRRSV VR2332 expressing the RFP-mCherry and GFP reporter genes, respectively. The reporter rEAV KY84-RFP mCherry virus was constructed by replacing F1 with a modified F1A-RFP mCherry (containing the RFP mCherry gene insert) and F1B. Subsequently, we performed CPER, transfected BHK-21 cells, and monitored virus recovery by live imaging of RFP-mCherry signal evident at 24 hpt and CPE at 72 hpt. The RFP mCherry signal continued to increase until the end of the transfection experiment at 72 hpt (Fig. 6a). The RFP mCherry signal of rEAV KY84-RFP mCherry P1 was expressed until 96 hpi and by 120 hpi the RFP mCherry signal declined due to 100% CPE affecting the cellular monolayer (Fig. S2). Finally, we performed immunostaining as indicated above, demonstrating colocalization of the RFP-mCherry signal with viral N and nsp1 (Fig. 6b, c).

**Fig. 7.**
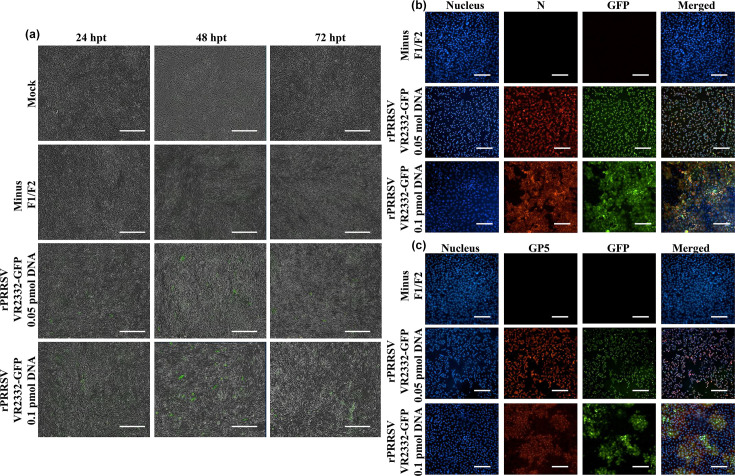
Live fluorescence imaging monitoring expression of GFP reporter gene in rPRRSV VR2332-GFP P0 in transfected BHK-21 from 24 to 72 hpt (**a**); magnification: 4×; scale: 400 µm. Recovery of the reporter virus in MARC-145 cells was confirmed by immunofluorescence using anti-N (**b**) and anti-GP5 antibodies (**c**). Magnification: 20×; scale: 100 µm.

To develop the reporter rPRRSV VR2332-GFP, the GFP gene was inserted between the stop codon of ORF1b and the start codon of ORF2a as described in the ‘Methods’ section. rPRRSV VR2332-GFP was efficiently recovered by transfecting the CPER product into BHK-21 cells, with GFP signal evident on live cell imaging starting at 24 hpt (Fig. 7a). As anticipated, no CPE was observed up to 72 hpt in BHK-21 cells. Following one passage in MARC-145 cells, CPE was detected at 72 hpi with a weak GFP signal evident at 48 hpi, which progressively increased through 96 hpi and declined at 120 hpi due to complete CPE on the monolayer (Fig. S3). Recovery of rPRRSV VR2332-GFP was confirmed by immunofluorescence, with colocalization of the GFP signal along with viral GP5 and N (Fig. 7b, c).

### Growth kinetics of infectious clones generated via CPER

**Fig. 8.**
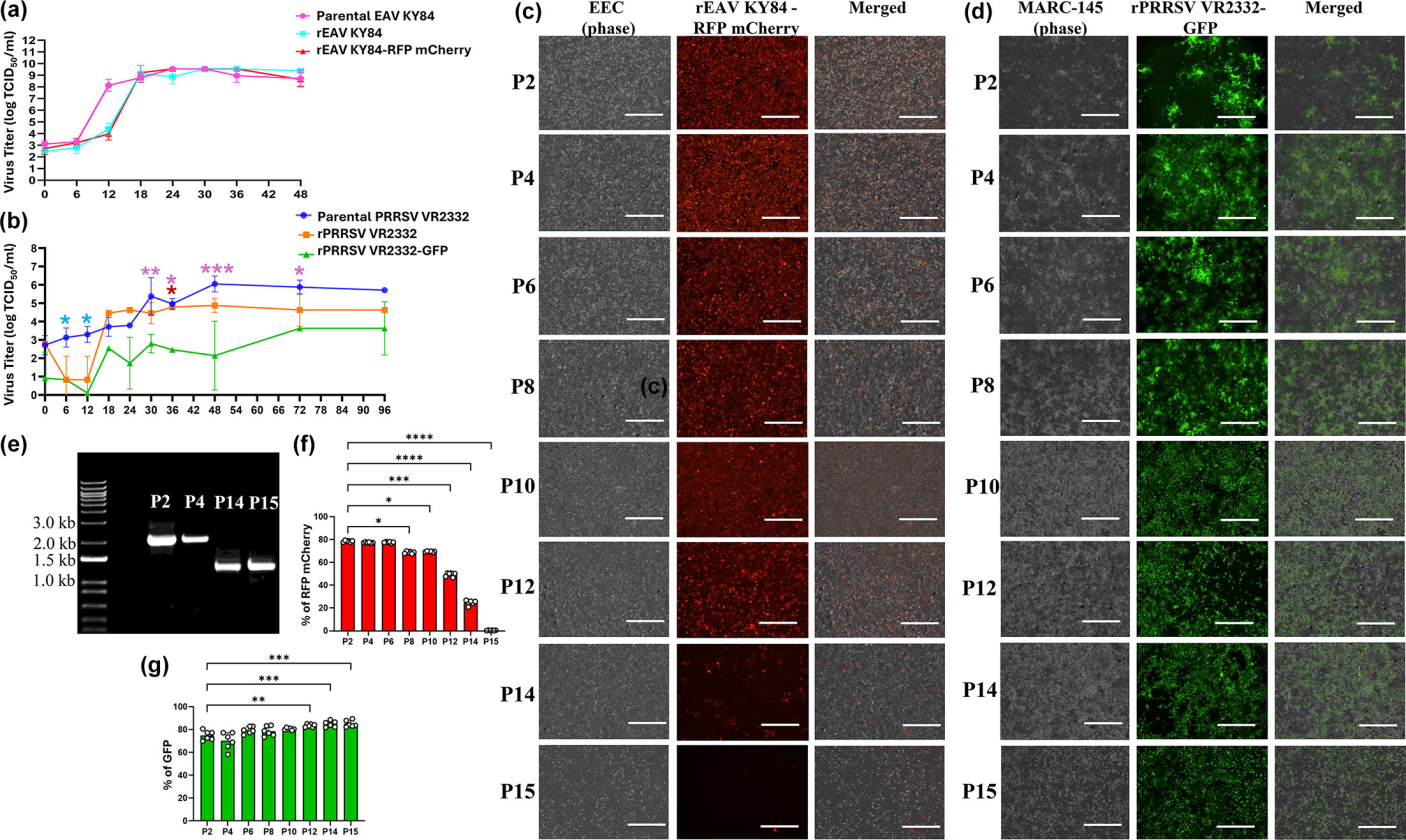
(**a**) Growth kinetics of rEAV KY84 (cyan) and rEAV KY84-RFP mCherry (red) compared to the parental EAV KY84 strain (magenta) in EEC cells (MOI=5). Each datapoint corresponds to triplicate wells and represents mean titre values±sd. (**b**) Growth kinetics of rPRRSV VR2332 (orange) and rPRRSV VR2332-GFP (green) compared to the parental PRRSV VR2332 strain (blue) in MARC-145 cells (MOI=5). Each datapoint corresponds to triplicate wells and represents mean titre values±sd. Light blue asterisks indicate statistically significant differences between rPRRSV VR2332 and parental PRRS VR2332, purple asterisks indicate statistically significant differences between rPRRSV VR2332-GFP and parental PRRS VR2332 and the red asterisk indicates statistically significant differences between rPRRSV VR2332 and rPRRSV VR2332-GFP (*P<0.05, **P≤0.01, ***P≤0.001). (**c**) Stability of RFP mCherry reporter gene following 15 serial passages of rEAV KY84-RFP mCherry in EEC cells, the expression of the RFP mCherry was stable until P6 and then, it decreased significantly to reach the lowest expression at P15. Magnification: 4×; scale: 400 µm. (**d**) Stability of GFP reporter gene following 15 serial passages of rPRRSV VR2332-GFP in MARC-145 cells. The expression of GFP was stable at least through P15. Magnification: 4×; scale: 400 µm. (**e**) Gel electrophoresis showing the amplification of F1A-RFP mCherry of rEAV KY84 reporter gene from viruses recovered at P2, P4, P14 and P15. The lower band size of P14 and P15 (1,264 bp) compared to the initial P2 and P4 (2,056 bp) indicates the in-frame deletion of the RFP mCherry insert (792 bp). (**f**) Stability of the RFP mCherry reporter gene following 15 serial passages of rEAV KY84-RFP mCherry in EEC. The reduction in RFP mCherry signal correlates with its deletion (*P<0.05, **P≤0.01, ***P≤0.001). (**g**) Stability of GFP reporter gene following 15 serial passages of rPRRSV VR2332 in MARC-145 cells. GFP signal is stable throughout the 15 serial passages (*P<0.05, **P≤0.01, ***P≤0.001).

We performed one-step growth kinetics for all CPER-generated infectious clones (rEAV KY84, rEAV KY84-RFP mCherry, rPRRSV VR2332 and rPRRSV VR2332-GFP) and compared them to their parental strains (EAV KY84 and PRRSV VR2332 strains). For CPER-generated rEAV KY84 and rEAV KY84-RFP mCherry, replication kinetics revealed that both CPER-generated recombinant viruses had comparable replication kinetics to their parental EAV KY84, with no significant differences in viral titres at all time points (Fig. 8a).

Similarly, the growth kinetics of rPRRSV VR2332 viruses were compared with those of parental PRRSV VR2332 over a 96 hpi time course in MARC-145 cells. The results showed that the parental PRRSV VR2332 and rPRRSV VR2332 had similar growth kinetics. Comparing the growth kinetics of the rPRRSV VR2332-GFP to the parental PRRSV VR2332 and rPRRSV VR2332, rPRRSV VR2332-GFP demonstrated comparatively reduced viral titres across multiple discrete time points, with up to a three-log_10_ difference (Fig. 8b). Taken together, the CPER-generated viruses in this study demonstrated replication kinetics equivalent to those of their parental strains, except for the rPRRSV VR2332-GFP reporter virus.

### CPER supports the generation of infectious clones of EAV KY84 and PRRSV VR2332 with high sequence identity

NGS was employed to conduct a comparative analysis of the viral genome sequences derived from CPER-generated rEAV and rPRRSV and their respective reporter counterparts. For rEAV KY84 and rEAV KY84-RFP mCherry, a 99.98% nucleotide identity to the parental EAV KY84 was determined with only one amino acid substitution in the rEAV KY84-RFP mCherry, as reported in Table 3. For rPRRSV VR2332, a 99.95% nucleotide identity was determined compared to the parental strain, while rPRRSV VR2332-GFP showed a 99.90% nucleotide identity. Only three and five non-synonymous mutations were detected in rPRRSV VR2332 and rPRRSV VR2332-GFP, respectively (Table 3). The NGS findings collectively underscore the precision and utility of the CPER methodology as a strategy for the generation of arterivirus infectious clones that significantly reduce sequence errors and increase fidelity.

**Table 3.** Comparative full-length genome sequence analysis of parental (EAV KY84 and PRRSV VR2332) and CPER-generated infectious clones (rEAV KY84, rEAV KY84-RFP mCherry, rPRRSV VR2332 and rPRRSV VR2332-GFP)

ORF	Gene name	Nucleotide position	Nucleotide sequence	Amino acid	Nucleotide sequence	Amino acid	Synonymous (S) or non-synonymous (NS)
			**Parental EAV KY84**		**rEAV KY84**		
ORF5	GP5	11,502	TTA	L	TTG	L	S
			**Parental EAV KY84**		**rEAV KY84-RFP mCherry**		
ORF1a	nsp7	4,616	GAT	D	GAC	D	S
ORF4	GP4	10,959	GAA	E	GGA	G	NS
ORF7	N	12,582	TAC	Y	TAT	Y	S
			**Parental PRRSV VR2332**		**rPRRSV VR2332**		
ORF1a	nsp5	6,675^*^	CCG	P	CTG	L	NS
ORF1b	nsp11	11,067	AAC	N	AAT	N	S
ORF1b	nsp11	11,328^*^	CAC	H	CAT	H	S
ORF1b	nsp11	11,373^*^	CAT	H	CAC	H	S
ORF2a	GP2a	12,364^*^	ATG	M	ATT	I	NS
ORF3	GP3	13,012^*^	GGT	G	AGT	S	NS
			**Parental PRRSV VR2332**		**rPRRSV VR2332-GFP**		
ORF1a	nsp1α	391	ACT	T	ACC	T	S
ORF1a	nsp1β	1,108	CTA	L	CTG	L	S
ORF1a	nsp3	4,945	GCT	A	GCC	A	S
ORF1a	nsp3	5,098	AGA	R	AGG	R	S
ORF1b	nsp9	8,262	CCG	P	CCA	P	S
ORF1b	nsp9	9,238	TCT	S	ACT	T	NS
ORF1b	nsp10	10,071	CCC	P	CCT	P	S
ORF1b	nsp10	10,534	TAC	Y	CAC	H	NS
ORF1b	nsp11	11,067	AAC	N	AAT	N	S
ORF1b	nsp11	11,082	CCA	P	CCC	P	S

*This mutation was detected in both CPER-generated viruses: rPRRSV VR2332 and rPRRSV VR2332-GFP.

### Stability of rEAV KY84-RFP mCherry and rPRRSV VR2332-GFP reporter viruses

The stability of RFP mCherry expression within ORF1a was investigated by serial passage of rEAV KY84-RFP mCherry in EECs. Expression of the RFP-mCherry reporter gene remained stable through the initial passages (P2 to P6) with an average of 77.93%±0.55%. Its expression was significantly reduced between P8 and P10 (69.19%±0.26%), with further reduction in signal to 49.38%±1.73% at P12, 24.80%±2.0% at P14 and minimal (0.43%±0.20%) at P15. This loss of expression was correlated with an in-frame deletion of the reporter gene sequence (Fig. 8c–f). By P16, the expression of RFP mCherry had completely ceased (data not shown). On the other hand, rPRRSV VR2332-GFP expression was relatively stable for at least 15 serial passages, maintaining a consistent expression level ranging from 75–85%±3.3% across all passages evaluated (Fig. 8d, g).

## Discussion

EAV and PRRSV are both members of the family *Arteriviridae*, a group of enveloped, positive-stranded RNA viruses within the order *Nidovirales*. While EAV primarily infects horses, PRRSV infects swine. In both cases, the diseases they cause have reproductive, respiratory and systemic implications for the infected host, and both lead to the establishment of persistent infections. Despite their distinct host specificities, the shared taxonomic classification of EAV and PRRSV highlights commonalities in their genome organization, replication strategies and interactions with the host [62,63].

Reverse genetic systems for RNA viruses have been widely implemented to systematically study viral genomes, facilitating research on viral gene structure, function, pathogenesis and virus–host interactions [35]. These methods have historically been based on manipulating the viral genome in a bacterial host*,* offering advantages for genetic modifications and the propagation of viral DNA as cDNA copies. However, they entail notable disadvantages, including time-consuming cloning procedures, as rescuing infectious viruses from these constructs often requires multiple steps and greater effort. Moreover, these constructs have the potential to cause genetic instability during bacterial replication, leading to mutations or rearrangements in the viral genome [39,64, 65]. The development of CPER as a versatile, fast and high-fidelity approach has provided a more streamlined and efficient alternative for generating infectious cDNA clones, thereby bypassing limitations associated with traditional BAC- and plasmid-based methods and improving virus rescue efficiency [40,42, 44, 66]. The goal of our study was to implement this technique as proof of concept for generating infectious clones of the arteriviruses, EAV KY84 and PRRSV VR2332 strains. Using this method, rEAV KY84 and rPRRSV VR2332 were recovered in BHK-21 cells at 2 and 4 days post-transfection, respectively, and the whole procedure, including overlapping cDNA fragments amplification, transfection and confirmation of the recovered virus, can be completed in less than 10 days (Fig. 1c). The CPER approach has demonstrated increased fidelity by significantly reducing the possibility of polymerase-induced mutations. This level of accuracy is highly desirable and essential for precisely assessing the functional impact of defined mutations and for developing stable vaccine candidates. In this study, we showed that CPER-generated arteriviruses have high sequence identity with their parental strains. However, we acknowledge that a very small percentage of polymerase-induced mutations can still arise, as shown in this study; therefore, future work to identify other high-fidelity polymerases that can be used to reduce this error rate is warranted.

Proper design of the overlapping cDNA fragments is critical for a successful CPER reaction. We have determined the number of fragments empirically based on prior CPER protocols used for assembly of the SARS-CoV-2 genome and modified based on the Tm of the overlapping regions. We have observed that the assembly of the SARS-CoV-2 genome, which is ~30 kb in length, in previous studies typically utilized 6 [40] to 10 [42,43] overlapping cDNA fragments. In this study, we attempted to apply this genome length/fragment number ratio as a fundamental criterion for fragmentation of the arteriviral genome. Hence, the ~15 kb PRRSV genome has been successfully fragmented into five fragments, whereas the ~12 kb EAV genome has been successfully fragmented into three fragments. The optimization of these fragmentation patterns is inherently an iterative process characterized by trials and errors. From the author’s point of view, successful CPER implementation relies on several critical factors during designing of the overlapped cDNA fragments: each fragment should ideally be less than 5 kb in length, the melting temperature of the designed overlapping cDNA fragments should be close to each other (50–60 °C), the melting temperature of the designed primers, and PCR amplification must yield high-purity bands to ensure reliable concentrations following the purification. These parameters are essential for maintaining the integrity of the assembly process, as the fidelity of the final construct is contingent upon the quality of the overlapping cDNA templates.

Initially, in CPER-based bacterial cloning, the linker segment is often a restriction-digested or PCR-generated linearized plasmid [67]. Subsequently, the linker design was adapted for the generation of infectious SARS-CoV-2 clones. This linker includes the viral 3′ UTR and a self-cleaving HDVr, which is essential for producing an authentic 3′ terminus of the viral RNA. In addition, the construct incorporates an SV40 poly(A) signal to promote efficient transcription termination, a spacer sequence to separate functional modules, a CMV promoter to enable *in vivo* transcription of viral RNA by cellular RNA polymerase II and the SARS-CoV-2 5′ UTR [40]. Liu and Gack further modified the linker design by using BGH poly(A) instead of SV40 poly(A), and by incorporating a functional Pol II transcriptional pause signal from the human *α*2-globin gene in place of the original spacer sequence located between the poly(A) signal and the CMV enhancer/promoter. This element ensures efficient Pol II termination and minimizes readthrough in the linker region, which could otherwise disrupt ribozyme processing at the 3′ end of the transcript or interfere with proper initiation of new transcription [43]. This modified linker was used in the present study to design the pEAV and pPRRSV linkers. The versatility of the CPER linker was further demonstrated in a recent study in which an RNA polymerase I (Pol I) promoter and terminator were used in place of the conventional Pol II promoter and poly(A) signal. This modification led to ~a 100-fold increase in viral output for viruses that rely on internal ribosome entry site (IRES)-mediated translation, including hepatitis C virus, bovine viral diarrhoea virus, hepatitis A virus and encephalomyocarditis virus. The use of Pol I transcriptional elements ensures that the resulting viral RNA transcripts possess authentic 5′ and 3′ termini without extraneous nucleotides or cap structures, thereby avoiding interference with IRES-mediated translation [68].

Genome size does not appear to impose a limitation on the CPER methodology. In the present study, CPER successfully enabled the recovery of infectious clones of EAV KY84 and PRRSV VR2332, with genome lengths of 12,704 bp and 15,451 bp, respectively. Moreover, other studies have applied CPER to generate infectious clones of RNA viruses with substantially larger genomes, including SARS-CoV-2 (~30 kb) [40,42, 43, 45] and the insect-specific Casuarina virus (~20 kb) [40]. The flexibility that this method provides helps study emerging viral variants, where rapid and easy introduction of specific mutations (e.g. point mutations, deletions or insertions) is required to understand their impact on replication, virulence, transmissibility, pathogenicity and immune evasion. In previous studies, CPER was successfully utilized to study SARS-CoV-2 variants, for example, by introducing specific naturally-occurring mutations (e.g. D614G into the spike protein) to understand virus pathogenesis and transmission [40,42]. In addition, CPER has been used to generate chimeric recombinant SARS-CoV-2 encoding the S gene of Omicron (BA.1 lineage) in the backbone of an ancestral SARS-CoV-2 isolate to determine how the spike protein contributes to SARS-CoV-2 pathogenicity [45]. The utility of this platform extends to the development of reporter viruses to investigate replication dynamics exemplified by the generation of a SARS-CoV-2 strain expressing the ZsGreen fluorescent protein [40] or GFP gene [42,43]. Beyond the *Coronaviridae* family, CPER was implemented in arthritogenic alphavirus Ross River virus (RRV) to elucidate the role of specific amino acids within the E3/E2 furin cleavage site in RRV replication, as well as to map the critical interactions between RRV and the host cell receptor MXRA8 [40]. For flaviviruses, a chimeric Zika virus was generated, using CPER, based on the Chaoyang virus backbone by substituting its prME with that of the Zika virus to study virus tropism in mosquitoes (C6/36) as well as vertebrate cells (Vero cells) [66]. Furthermore, landmark development has occurred in implementing the CPER method to negative-stranded RNA viruses (Mononegaviruses), generating an infectious clone of RSV by co-expression of the full-length RSV antigenome cDNA with plasmids encoding the nucleoprotein (N), the phosphoprotein (P), the large polymerase protein (L) and M2 (ORF1) proteins essential for viral replication [46] and, similarly, for rabies virus (RABV). In the latter, mutants carrying point mutations, exogenous reporter genes or chimeric G genes have been generated to study their neurovirulence and the function of the glycoprotein (G) in viral entry [69]. CPER has also been used to support the generation of reverse genetics-based vaccines, and it has been utilized to produce the Japanese encephalitis virus vaccine strain SA14-14-2 as a backbone for constructing chimeric flavivirus vaccine candidates [66]. Hence, we conclude that CPER provides considerable flexibility for manipulating viral genome sequences through PCR-based mutagenesis. Simply, if multiple mutations are to be studied, they can be incorporated into a single fragment, which can be generated synthetically and incorporated into the CPER reaction.

In the current study, our CPER-generated reporter virus, rEAV KY84-RFP mCherry, demonstrated stability similar to the previously developed reporter EAV virulent Bucyrus strain, in which the mCherry expression was stable for at least seven serial passages, after which deletion of the reporter gene mCherry-2A occurred, becoming undetectable by P12 to P16 [50]. On the other hand, our rPRRSV VR2332-GFP strain showed a stability of GFP expression for at least 15 serial passages, which is in agreement with Pei and others who demonstrated the stability of the GFP expression in the North American PRRSV strain P129 up to 37 passages [51]. The differences in stability of the reporter constructs are likely related to the insertion site used in the viral genome. For EAV, the RFP-mCherry gene was inserted into ORF1a downstream of the nsp1/nsp2 cleavage junction, while for PRRSV, the GFP reporter gene was inserted into the short region between the stop codon of ORF1b and the start codon of ORF2a under TRS2. It was reported previously that the insertion of the GFP or RFP mCherry gene in-frame into or between the hypervariable regions to create nsp2-reporter fusion proteins allowed the successful production of recombinant reporter viruses, but not phenotypically stable after several cell culture passages [50,59, 70, 71]. In contrast, when the reporter gene is inserted as a separate transcription unit, such as the strategy used for PRRSV, this results in an additional sgRNA without altering the coding sequence of a viral gene and minimizing the possibility of recombination and deletion of the reporter gene [51]. Such differences in reporter gene stability were faithfully recapitulated in the CPER-recovered reporter viruses as previously described using conventional reverse genetic platforms for EAV and PRRSV.

An additional advantage of the CPER assay is its ability to generate recombinant viruses from clinical specimens without prior virus isolation. Amarilla and others were able to recover human norovirus (HuNoV) (~7.5 kb) from a patient’s faecal sample [40]. Additionally, recombinant bovine viral diarrhoea virus was successfully generated from the serum of persistently infected cattle using the modified CPER assay, and its replication properties were comparable to those of the original clinical isolate [68]. Therefore, CPER is considered an efficient reverse genetics system for generating replication-competent viruses from clinical samples, which is particularly useful when viruses from field samples are difficult to propagate in cell culture or during outbreaks when urgent interventions are required for crucial control.

In summary, we established a simple, rapid and versatile CPER-based reverse genetics platform for generating infectious clones of arteriviruses (EAV and PRRSV) and for efficiently introducing reporter genes. This system provides a powerful tool for studying viral gene functions and virus–host interactions.

## Conclusion

CPER offers several advantages over traditional restriction enzyme-based cloning approaches for generating infectious cDNA clones of RNA viruses. Unlike conventional methods that require stepwise cloning, ligation and propagation of full-length viral genomes in bacteria, which often lead to instability, recombination or toxicity, the CPER technique enables seamless assembly of overlapping viral genome fragments using high-fidelity PCR enzymes without reliance on restriction sites or extensive bacterial amplification. This approach markedly accelerates clone construction, reduces the incidence of unwanted mutations and preserves genome integrity, particularly for large or genetically unstable RNA viruses. CPER also provides greater flexibility for genome manipulation (i.e. reverse genetics), allowing rapid introduction of mutations, insertions or reporter genes directly at the fragment level. Overall, CPER streamlines reverse genetics workflows, enhances reproducibility and is exceptionally well-suited for studying emerging or difficult-to-clone RNA viruses compared with traditional cloning techniques and also helps to generate vectored viral vaccines.

## Supplementary material

10.1099/acmi.0.001181.v3 Uncited Supplementary Material 1.

## Abbreviations

BACs: bacterial artificial chromosomes
BGH: bovine growth hormone
BHK-21: baby hamster kidney-21 cells
CMV: cytomegalovirus
CPE: cytopathic effect
CPER: circular polymerase extension reaction
DMVs: double-membrane vesicles
EAV: equine arteritis virus
EAV KY84: equine arteritis virus KY84 strain
EECs: equine pulmonary artery endothelial cells
HDVr: hepatitis delta virus ribozyme
hpi: hours post-infection
hpt: hours post-transfection
IRES: internal ribosome entry site
MOI: multiplicity of infection
NFW: nuclease-free water
NGS: next-generation sequencing
nsp: non-structural protein
ORF: open reading frame
Pol I: polymerase I
Pol II: polymerase II
PRRSV: porcine reproductive and respiratory syndrome virus
PRRSV VR2332: porcine reproductive and respiratory syndrome virus VR2332 strain
rEAV: recombinant equine arteritis virus
RFP: red fluorescence protein
rPRRSV: recombinant porcine reproductive and respiratory syndrome virus
RRV: Ross River virus
RSV: respiratory syncytial virus
RT: room temperature
TCID_50_: tissue culture infective dose 50%
TRSs: transcription-regulating sequences
UTR: untranslated region
