# Supplementary material for: Reverse genetics approach for arteriviruses using circular polymerase extension reaction
Source: Access Microbiol. 2026 May 21;8(5):001181.v3. doi: 10.1099/acmi.0.001181.v3 (PMC13193255; doi:10.1099/acmi.0.001181.v3)
Supplement: Uncited Supplementary Material 1. [file acmi-8-01181-s001.pdf]

Supplementary figures and legends

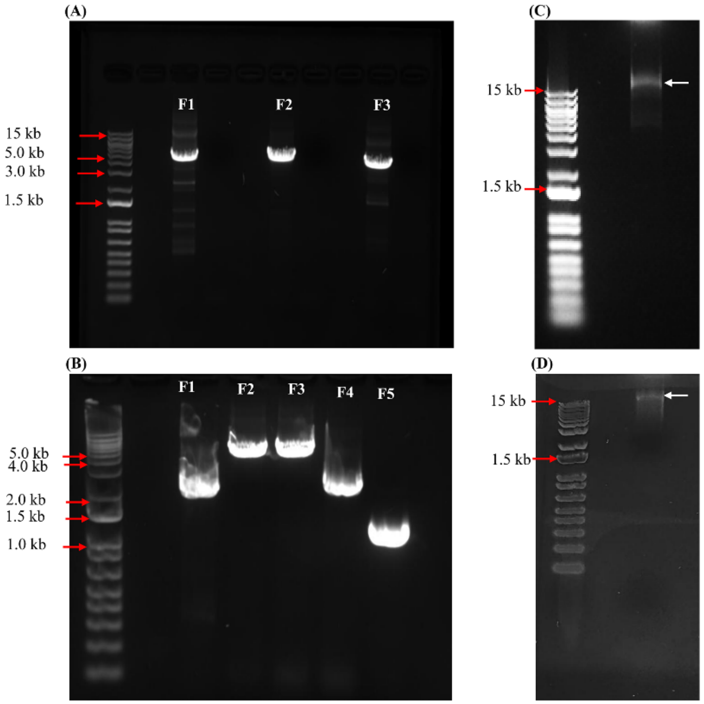

Supplementary Figure 1. High-fidelity PCR amplification and CPER reaction. Full-genome amplification of three overlapping fragments (F1 [4,300 bp], F2 [4,300 bp] and F3 [4,100 bp]) of EAV KY84 **(A)**, five overlapping fragments (F1 [2,200 bp], F2 [4,923 bp], F3 [4,923 bp], F4 [2,303 bp] and to F5 [1,086 bp]) of PRRSV VR2332 **(B)**, and a high molecular weight product (indicated by white arrow) obtained by circularization of the annealed genome amplified fragments with the pEAV KY84 linker **(C)** and pPRRSV VR2332 linker **(D)** using CPER .

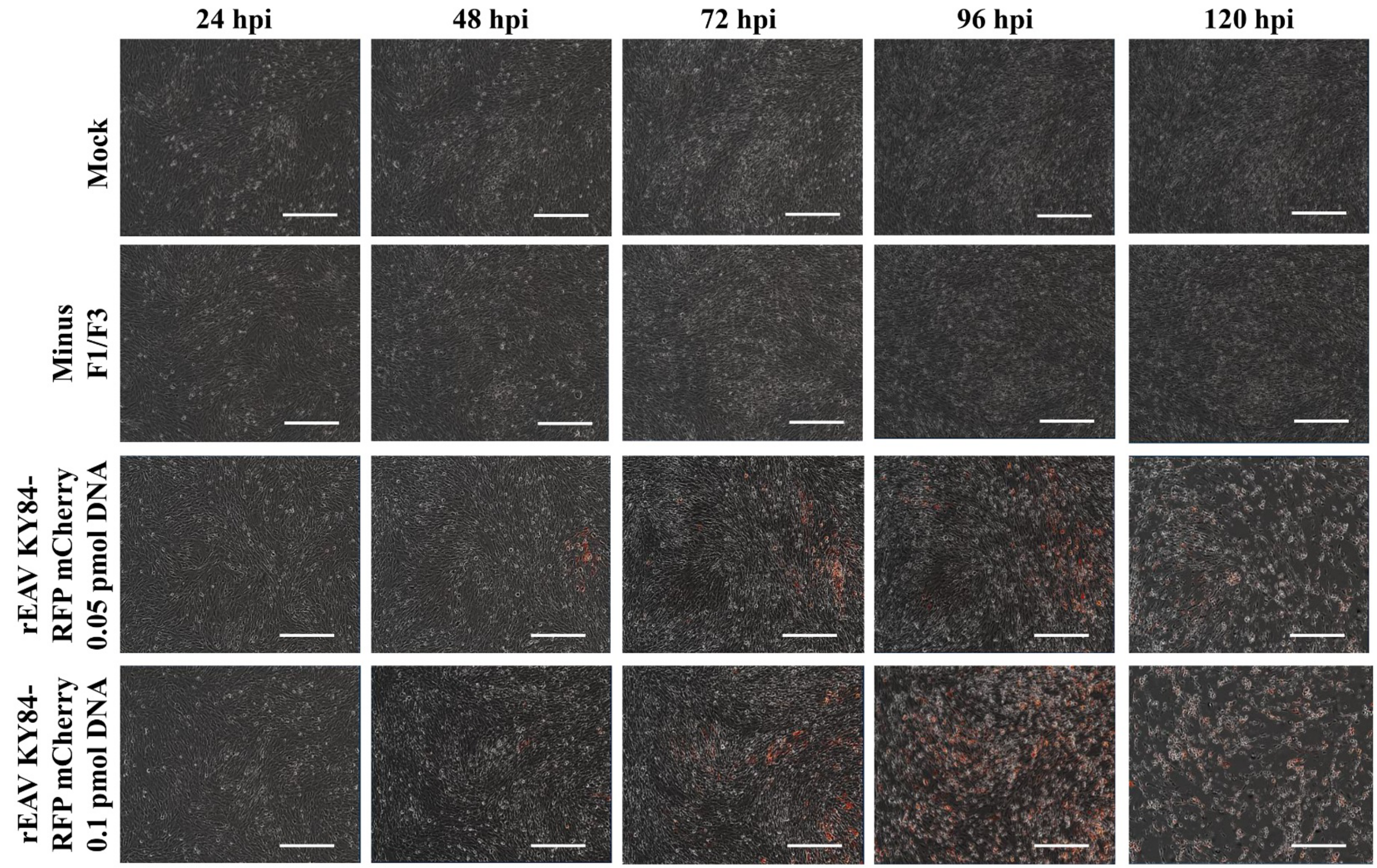

Supplementary Figure 2. Live fluorescence imaging demonstrates the expression of RFP mCherry reporter gene in rEAV KY84-RFP mCherry P1 in infected BHK-21 from 24 to 120 hpi. Magnification: 4×; scale: 400 μm.

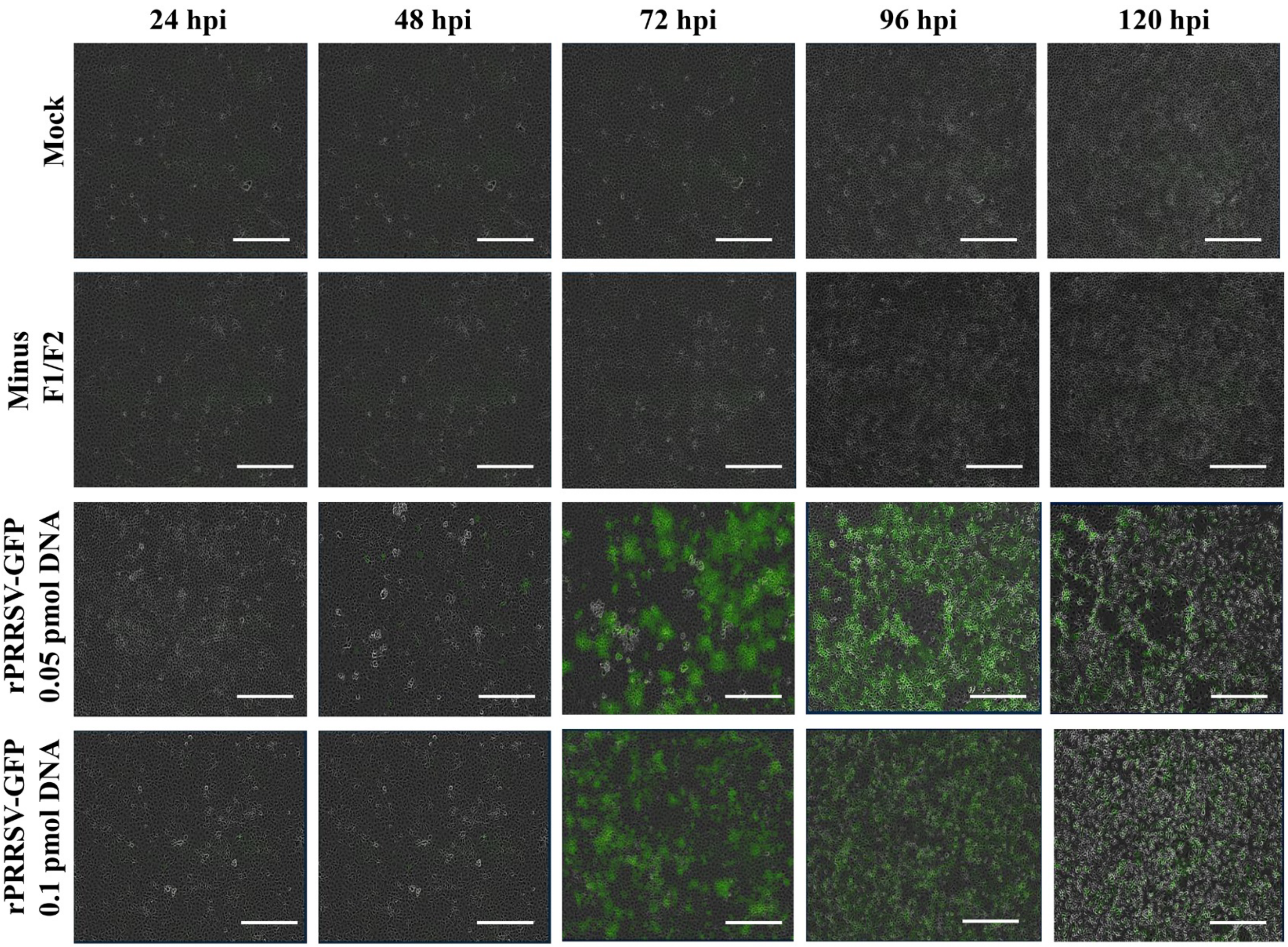

Supplementary Figure 3. Live fluorescence imaging demonstrates the expression of GFP reporter gene in rPRRSV VR2332-GFP P1 in infected MARC-145 cells from 24 to 120 hpi. Magnification: 4×; scale: 400 μm.
